# The GH10 and GH48 dual-functional catalytic domains from a multimodular glycoside hydrolase synergize in hydrolyzing both cellulose and xylan

**DOI:** 10.1186/s13068-019-1617-2

**Published:** 2019-12-03

**Authors:** Yindi Chu, Zhenzhen Hao, Kaikai Wang, Tao Tu, Huoqing Huang, Yuan Wang, Ying Guo Bai, Yaru Wang, Huiying Luo, Bin Yao, Xiaoyun Su

**Affiliations:** 1 Key Laboratory for Feed Biotechnology of the Ministry of Agriculture, Feed Research Institute, Chinese Academy of Agricultural Sciences, No. 12 South Zhongguancun Street, Beijing, 100081 China; 2 Department of Microbiology and Parasitology, Institute of Basic Medical Sciences, Chinese Academy of Medical Sciences and Peking Union Medical College, 5# Dong Dan San Tiao, Beijing, 100005 China

**Keywords:** *Caldicellulosiruptor bescii*, Cellulase, Xylanase, Synergy, GH10, GH48, Multimodular, Bifunctional, Biofuel

## Abstract

**Background:**

Regarding plant cell wall polysaccharides degradation, multimodular glycoside hydrolases (GHs) with two catalytic domains separated by one or multiple carbohydrate-binding domains are rare in nature. This special mode of domain organization endows the *Caldicellulosiruptor bescii* CelA (GH9-CBM3c-CBM3b-CBM3b-GH48) remarkably high efficiency in hydrolyzing cellulose. *Cb*Xyn10C/Cel48B from the same bacterium is also such an enzyme which has, however, evolved to target both xylan and cellulose. Intriguingly, the GH10 endoxylanase and GH48 cellobiohydrolase domains are both dual functional, raising the question if they can act synergistically in hydrolyzing cellulose and xylan, the two major components of plant cell wall.

**Results:**

In this study, we discovered that *Cb*Xyn10C and *Cb*Cel48B, which stood for the N- and C-terminal catalytic domains, respectively, cooperatively released much more cellobiose and cellotriose from cellulose. In addition, they displayed intramolecular synergy but only at the early stage of xylan hydrolysis by generating higher amounts of xylooligosaccharides including xylotriose, xylotetraose, and xylobiose. When complex lignocellulose corn straw was used as the substrate, the synergy was found only for cellulose but not xylan hydrolysis.

**Conclusion:**

This is the first report to reveal the synergy between a GH10 and a GH48 domain. The synergy discovered in this study is helpful for understanding how *C. bescii* captures energy from these recalcitrant plant cell wall polysaccharides. The insight also sheds light on designing robust and multi-functional enzymes for plant cell wall polysaccharides degradation.

## Background

Degradation of plant cell wall polysaccharides (PCWP, mainly consisting of cellulose and xylan) by microbial glycoside hydrolases (GHs) is an essential step towards carbon cycling on earth [1]. The knowledge about such an enzymatic hydrolysis of PCWP has long been harnessed in treating lignocellulose-containing food, feed, and textile, and more recently, in developing biofuels [2, 3]. However, up-to-date the cost of cellulase and xylanase remains as a bottleneck for the biofuel industry [4]. Improving the productivity of microbial workhorses such as the saprophytic fungus *Trichoderma reesei* for producing cellulase is helpful for cost saving [5, 6]. Besides, engineering cellulase and xylanase for higher catalytic efficiency is also being intensively studied, with the employment of new degradation strategies as a straightforward and effective way. As an example, supplementing with lytic polysaccharide monooxygenases (LPMOs) acting in an oxidative manner in a canonical hydrolytic cellulase blend boosts cellulose degradation [7].

Most naturally occurring enzymes degrading cellulose and xylan can be grouped into two categories, i.e., free enzymes or cellulosomes, with the former represented by the enzymes from aerobic fungi (such as *T. reesei*) and the latter by those from some anaerobic bacteria (such as *Clostridium thermocellum*), respectively [1, 8]. Multimodular glycoside hydrolases with two catalytic domains separated by one or multiple carbohydrate-binding modules (CBMs) are rare in nature and appear to be an intermediate paradigm existing between free enzymes and cellulosomes [9–11]. CelA [12] (or *Cb*Cel9A/Cel48A [13]) from a thermophilic bacterium *Caldicellulosiruptor bescii* is such an enzyme with this special mode of domain organization by having N-terminal GH9 and C-terminal GH48 catalytic domains separated by three family 3 cellulose-binding CBMs (GH9-CBM3c-CBM3b-CBM3b-GH48). Impressively, this enzyme displays a high efficiency in hydrolyzing crystalline cellulose [12, 14, 15]. It is noticed that, in this bacterium, there are five more multimodular GHs all sharing a similar domain architecture, majorly differing in their N- and C-terminal catalytic domains [16].

Among the six multimodular GHs in *C. bescii*, *Cb*Xyn10C/Cel48B [17] (or CelC [18]) is intriguing in that it has a nearly identical domain organization pattern, as well as amino acid sequence, to that of CelA [19]. The most prominent difference is that the N-terminal GH9-CBM3c domains in CelA are replaced by a GH10 catalytic domain in *Cb*Xyn10C/Cel48B. Strong intermolecular synergy in hydrolyzing crystalline cellulose has been observed when combining some of these multimodular enzymes (such as CelA and *Cb*Xyn10C/Cel48B), which is believed to be important for cellulose utilization by *C. bescii* [18]. However, until now the underlying mechanism about such a synergy has not been fully elucidated. In our previous studies, we discovered that, while the GH10 domain is a bona fide xylanase, it displays a remarkable side endo-cleaving activity for crystalline cellulose [17, 20]. This makes *Cb*Xyn10C/Cel48B extremely similar to CelA in domain organization by having an endoglucanase in the N-terminus and an exo-glucanase in the C-terminus, respectively. Therefore, it is tempting to hypothesize that the two catalytic domains within the same polypeptide may synergize in hydrolyzing cellulose. Moreover, Cel48A (the GH48 cellulase domain in CelA) has been reported to also have promiscuity in hydrolyzing xylan [14]. Since the amino acid sequences of Cel48A and Cel48B are identical, this further raises the question if *Cb*Xyn10C and *Cb*Cel48B can act synergistically in hydrolyzing xylan, as well. The knowledge about the synergistic effect of these domains in hydrolyzing cellulose and xylan would be helpful for understanding how *C. bescii* captures energy from these recalcitrant polysaccharides. A previous truncation study of Cdan_2053 (GH10-CBM3-GH12-GH48) from *Caldicellulosiruptor danielii* [18] and the above-addressed cooperation among specific combinations of multimodular *C. bescii* full-length enzymes [19, 21] have provided clues but not definitive evidence for the proposed synergy between the GH10 and GH48 catalytic domains. Therefore, hydrolysis of cellulose and xylan by *Cb*Xyn10C and *Cb*Cel48B catalytic domains was investigated in this study and the acquired knowledge will help us understand the underlying mechanism of synergy between the *C. bescii* multimodular, dual catalytic domain enzymes.

## Results and discussion

### Expression of three truncation mutants of *Cb*Xyn10C/Cel48B bearing one or both of the GH10/GH48 catalytic domains

*Caldicellulosiruptor bescii* uses predominantly multimodular enzymes to degrade cellulose, which is prototyped by CelA with one GH9 and one GH48 catalytic domain tethered in a single polypeptide. This has been proposed to be an intermediate between free enzyme and supramolecular cellulosome paradigms [14, 22]. Similar to CelA, during growth of *C. bescii* on cellulose, *Cb*Xyn10C/Cel48B (or Athe_1857) is also abundantly secreted by *C. bescii* [10, 23]. This points to its possibly important role involved in cellulose depolymerization. Instructively, its domain organization and amino acid sequence are extremely similar to CelA, with the major difference existent only in the N-terminus [17]. Since our previous studies have demonstrated that *Cb*Xyn10C has non-negligible activity on crystalline cellulose, it is therefore hypothesized that *Cb*Xyn10C may synergize with the C-terminal GH48 cellobiohydrolase to degrade cellulose.

**Fig. 1:**
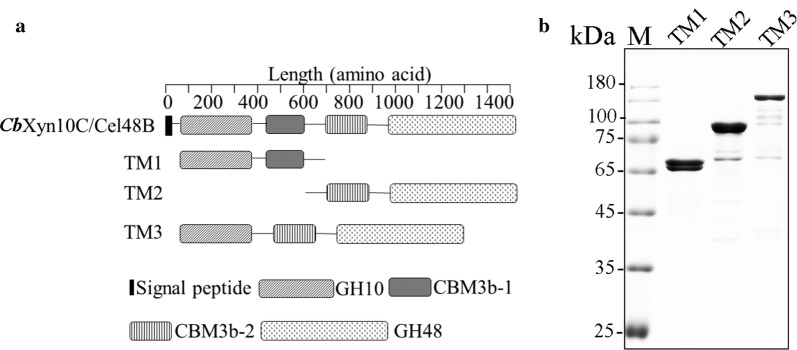
Preparation of truncation mutants of *Cb*Xyn10C/Cel48B. **a** Schematic diagram of the truncation mutants. **b** SDS-PAGE analysis of purified TM1, TM2, and TM3. Lane M, protein molecular mass marker

Obtaining truncation mutants with either of the GH10 or GH48 catalytic domains, or both, is the prerequisite to understand if these two domains can act synergistically in degrading plant cell wall polysaccharide in *Cb*Xyn10C/Cel48B. Therefore, the N-terminal GH10 catalytic domain appended with a CBM3b (GH10-CBM3b-1), herein termed TM1, and the C-terminal GH48 catalytic domain linked to a CBM3b (CBM3b-2-GH48, termed TM2) were cloned and expressed in *E. coli* (Fig. 1a). These truncation mutants were selected to represent the single catalytic domain enzymes from other microbial systems, with the CBM3b reported to assist hydrolysis of cellulose [17]. Note that CBM3b-1 and CBM3b-2 have nearly identical amino acid sequences (> 99% identity). The overexpressed recombinant TM1 and TM2 enzymes were purified by using immobilized metal affinity chromatography followed by ion exchange. Unfortunately, the expression level of full-length *Cb*Xyn10C/Cel48B was too low, preventing us from purifying the holoenzyme. However, a truncation mutant of *Cb*Xyn10C/Cel48B which has the two catalytic domains separated by one CBM3b (i.e., GH10-CBM3b-2-GH48, herein termed TM3) can be expressed and purified (Fig. 1b). Since this domain organization is similar to that of the wild-type enzyme, this construct can be regarded as a shortened mimic of the full-length multimodular enzyme *Cb*Xyn10C/Cel48B and thus selected for further analyses. Therefore, these three proteins were compared for their activities on cellulose and xylan.

### Synergistic hydrolysis of filter paper cellulose by the GH10 and GH48 catalytic modules

**Fig. 2:**
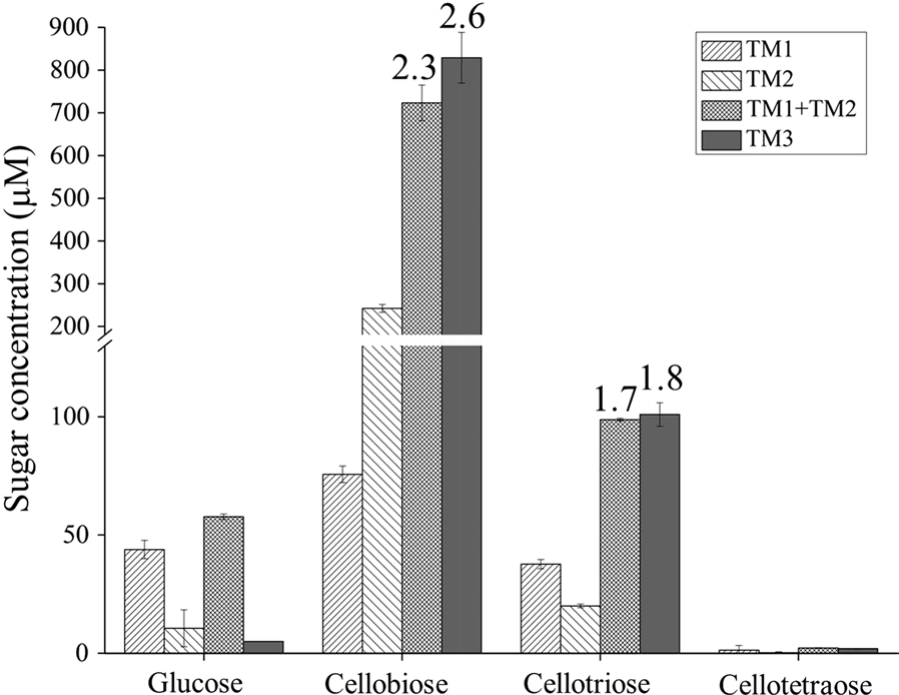
The GH10 and GH48 domains synergize in hydrolyzing filter paper, a crystalline form of cellulose. Filter paper was incubated with the truncation mutants at 75 °C, pH 6.5 (McIlvaine buffer) for 5 h and the reaction products were analyzed by HPAEC–PAD. The degrees of synergy are indicated, which were calculated by dividing the amount of a sugar of TM1 + TM2 or TM3 by the sum of the sugar released by TM1 and TM2 separately

We first used the DNS method to measure the specific activity of the three truncation mutants on cellulosic substrates. The reaction conditions were set as 75 °C for cellulose (to minimize loss of enzyme activity in the incubation) and 85 °C for xylan (to maximize the enzyme activity of xylanase in the short period of incubation) in a pH 6.5 McIlvaine buffer (in accordance with the optimal pH of *Cb*Xyn10C) [17]. In the long incubation at 75 °C, no loss of enzyme activity was observed for TM1 and TM3, while only slight reduction of enzyme activity (10%) was monitored for TM2 (Additional file 1). As expected, TM3 displayed activity on both xylan and cellulose (CMC and filter paper, representing soluble and insoluble cellulose, respectively), in accordance with its GH10 xylanase and GH48 cellulase domains, respectively (Table 1). TM1 also had activity on the two cellulosic substrates, indicating that the GH10 domain encodes a bifunctional xylanase/cellulase, as reported earlier [17, 20]. TM2, which bears the GH48 catalytic domain, had activity on filter paper, CMC, and xylan. At the first glance, the reducing sugar assay as determined by the DNS method did not indicate any significant synergy of the GH10 and GH48 catalytic modules on cellulose, either intermolecularly (by mixing TM1 and TM2 together) or intramolecularly (by acting of the TM3) (Table 1), as determined for the cellulase specific activity. However, it was noticed that the hydrolysis products were heterogeneous and contained multiple sugars. Through HPAEC–PAD analysis, *Cb*Xyn10C was determined to produce glucose to cellohexaose [17], while *Cb*Cel48B released glucose to cellotriose from filter paper [13]. These sugars could react differently with the DNS reagent [24]. Therefore, cellulose hydrolysis with these enzymes was carried out under the same conditions for 5 h when the reaction entered a plateau phase [17]. HPAEC–PAD was further used to determine the components in the filter paper hydrolysis products. This analysis indicated that, although there was still no synergistic effect for glucose liberation, significant synergy was observed for release of cellobiose and cellotriose, with degrees of synergy (DoS) of 2.3 and 1.7, respectively. for TM1 + TM2 binary mixture (Fig. 2). The DoS in releasing cellobiose and cellotriose was 2.6 and 1.8, respectively (Fig. 2). Adding the sugars together, the synergy was 2.0 for TM1 + TM2 and 2.2 for TM3, respectively. Thus integrating the GH10 and GH48 modules in a single polypeptide did not result into elevated synergy for the liberation of cellobiose and cellotriose.

**Table 1:** Substrate specificity of TM1, TM2, and TM3

	Specific activity (μmol/min/μmol of enzyme)		
	Filter paper	CMC	Xylan
TM1	7.1 ± 0.3	14.0 ± 0.4	10,276 ± 191
TM2	3.3 ± 0.1	4.6 ± 0.2	4.5 ± 0.4
TM1 + TM2	8.2 ± 0.6	15.8 ± 1.1	10,726 ± 106
TM3	11.5 ± 0.1	21.9 ± 2.6	17,331 ± 286

Values are represented as means ± standard deviations from three independent experiments

Similar to the domain structure of TM3, CelYZ is an artificial enzyme generated by fusing the *Clostridium stercorarium* CelY GH48-CBM(B,C) (exo-acting) with the CelZ GH9-CBM(Cʹ) (endo-acting) domains into a single polypeptide [22]. Therefore, although a further improvement of cellulase activity has been observed for the CelYZ fusion protein compared to the simple mixture of CelY GH48-CBM(B,C) and CelZ GH9-CBM(Cʹ), such an additional increment appeared to be not existing for TM3 in cellulose hydrolysis. It is hypothesized, however, with one more CBM3b, the full-length enzyme *Cb*Xyn10C/Cel48B should have higher affinity for crystalline cellulose than TM3. Since degradation of crystalline cellulose by cellulase is at the interface of liquid/solid phases, the consequently higher local concentration of *Cb*Xyn10C/Cel48B at this interface can likely facilitate cooperative action of the GH48/GH10 domains in cellulose hydrolysis and therefore, an intramolecular synergy might be observed for the full-length enzyme. This hypothesis undoubtedly requires further experimental analysis. Interestingly, while TM1 + TM2 released 57 μM of glucose, TM3 only produced 5 μM of glucose, indicating that the hydrolysis pattern changed for TM3, which was also observed for CelYZ. This suggests that the presence of two catalytic domains in the same polypeptide may affect each other and thus have an impact on their overall functions.

Although the ratio of glucose:cellobiose:cellotriose (1:166:20) for TM3 was much different from that of TM1 + TM2 (1:12.5:1.7) and those of *Cb*Xyn10C and *Cb*Cel48B acting alone (1:1.7:0.9 and 1:23:1.9, respectively) (Fig. 2), in the cases of both inter- and intramolecular interactions, the GH10 and GH48 acted together to produce predominantly cellobiose with nearly identical high concentrations. This indicated that cellotriose, and perhaps longer cellooligosaccharides produced by *Cb*Xyn10C during cellulose hydrolysis as well, was quickly degraded by *Cb*Cel48B majorly into cellobiose. The preferred release of cellobiose by cooperation of *Cb*Xyn10C and *Cb*Cel48B from cellulose is of physiological significance since cellobiose is among the best substrates to support growth of *C. bescii*, on which a large amount of cellulase is expressed [25]. Similarly, CelA (*Cb*Cel9A/Cel48A) also produces a large amount of cellobiose [13]. However, CelA produces considerable amounts of glucose from filter paper in addition to cellobiose [13]. In addition, the cooperation between *Cb*Xyn10C and *Cb*Cel48B in cellulose hydrolysis can, at least partially, account for the observed synergy between *Cb*Xyn10C/Cel48B and CelA [18]. Since the Cel48A and Cel48B domains are identical, the GH10 domain in *Cb*Xyn10C/Cel48B should be able to synergize intermolecularly with the CelA GH48 domain, as demonstrated in Fig. 2. Therefore, in nature, the true extracellular cellulose hydrolysate profile tends to be a product mixture from single multimodular cellulase and their combinatorial action. Adding to this complexity, the cellular transmembrane transporters involved in cellooligosaccharides assimilation should also be taken into consideration, whose substrate specificity (i.e., preference for long or short cellooligosaccharides) and transporting efficiency will have a large impact on the hydrolysate profile. To the best knowledge of the authors, no *C. bescii* cellooligosaccharides transporters have been identified and biochemically characterized.

Although *Cb*Xyn10C has a specific activity of 5.4 μmol/min/μmol of enzyme on filter paper, comparable to those of other typical endoglucanases such as *Cb*Cel9B/Man5A (16.1 μmol/min/μmol of enzyme) [26], the GH12 CelA cellulase of *Thermotoga neapolitana* (3.2 μmol of sugar/min/μmol of enzyme) [27], and CelB of *Caldicellulosiruptor saccharolyticus* (1.8 μmol of sugar/min/μmol of enzyme) [28], its activities on soluble cellulose, e.g., sodium carboxymethyl cellulose, and amorphous cellulose (such as phosphoric acid-swollen cellulose) are relatively low (39 and 1.2 μmol of sugar/min/μmol of enzyme, respectively). Despite its apparently low activity with these commonly easy-to-digest cellulose substrates, *Cb*Xyn10C cooperates with *Cb*Cel48B to efficiently hydrolyze filter paper, which is instead a crystalline form of cellulose and more resistant to hydrolysis. The specific activity of TM3, a miniaturized mimic of the full-length enzyme *Cb*Xyn10C/Cel48B, exhibited a specific activity of 11.5 μmol of sugar/min/μmol of enzyme on filter paper, similar to that of CelA (13.1 μmol of sugar/min/μmol of enzyme) expressed in *E. coli* [13]. Previous truncation studies of *Cb*Cel9B/Man5A and *Cb*Xyn10C/Cel48B revealed that appending two CBM3bs to *Cb*Cel9B and *Cb*Xyn10C is beneficial for cellulose hydrolysis [17, 29]. Therefore, although the full-length enzyme was not obtained in this study, it is postulated that *Cb*Xyn10C/Cel48B may be superior to TM3. Considering that *Cb*Xyn10C has significant xylanase activity, *Cb*Xyn10C/Cel48B appears to be evolved to hydrolyze the intertwined cellulose/xylan polysaccharides in plant cell wall.

### Intramolecular synergy of the GH10 and GH48 catalytic modules in early hydrolysis of xylan

**Fig. 3:**
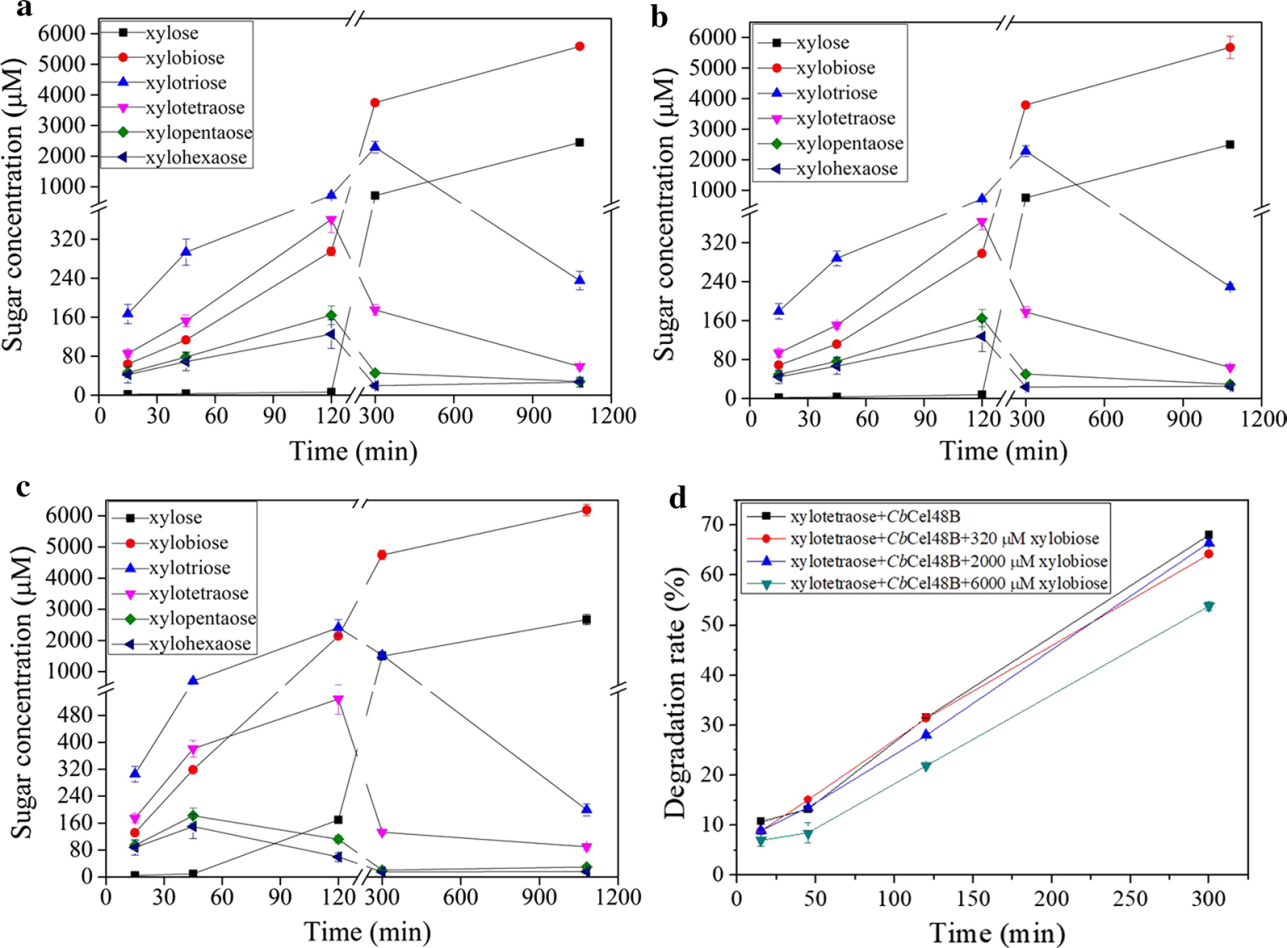
Time-course analysis of xylan hydrolysis by the truncation mutants. **a**–**c** Xylan hydrolysis by TM1 (**a**), TM1 + TM2 (**b**), and TM3 (**c**). **d** Hydrolysis of xylotetraose by *Cb*Cel48B was inhibited by increasing amounts of xylobiose. The hydrolysis was carried out at 75 °C in the McIlvaine buffer (pH 6.5) for 18 h and at different time intervals the samples were taken out for HPAEC–PAC analysis

Although the GH48 domain is a typical exocellulase, *C. bescii* Cel48A could also hydrolyze xylan [14]. Due to the identical amino acid sequence of the GH48 domain in *Cb*Xyn10C/Cel48B to that of Cel48A, we next determined if the GH10 and GH48 modules in *Cb*Xyn10C/Cel48B could act synergistically on xylan. The assay conditions were set as 75 °C (to minimize loss of enzyme activity in the 18 h of incubation) in the pH 6.5 McIlvaine buffer. Unlike cellulose hydrolysis, although mixing the GH10 and GH48 modules (TM1 + TM2) did not give any synergy, TM3 by itself displayed significant higher specific activity on xylan in the DNS measurement of reducing sugars (Table 1). Since the specific activity was determined in the early stage of xylan hydrolysis (10 min), we further carried out a time-course analysis of xylan hydrolysis and determined the components of the reaction products using HPAEC–PAD (Fig. 3). Although TM2 at high concentrations (1 μM and 2 μM) released significant amounts of xylose and xylooligosaccharides, TM2 at a low concentration (100 nM) liberated only minor concentrations of these sugars (Additional file 2). Therefore, the components in TM2-hydrolyzed products were negligible and therefore not presented. TM1, TM1 + TM2, and TM3 all produced xylose and xylooligosaccharides (xylobiose to xylohexaose). TM1 and TM1 + TM2 generated extremely similar hydrolysis patterns (Fig. 3a, b). Xylotriose was the dominant hydrolysis product from 15 to 45 min. Xylobiose and xylose began to accumulate from 120 min to 18 h, while the concentrations of the comparatively longer xylooligosaccharides (xylotriose to xylohexaose) dropped during this period. The three enzymes/enzyme combination released very similar concentrations of xylose to xylohexaose at the end of reactions (Fig. 3a–c). However, from 15 to 120 min which was in the early stage of hydrolysis, the concentrations of xylobiose, xylotriose, and xylotetraose released by TM3 rapidly increased to 2141 μM, 2415 μM, and 529 μM, respectively, which were much higher than those released by TM1 (xylobiose, 295 μM; xylotriose, 716 μM; xylotetraose, 361 μM) or TM1 + TM2 (xylobiose, 297 μM; xylotriose, 718 μM; xylotetraose: 364 μM) at 120 min (Fig. 3a–c). Release of xylose was also faster by TM3 (compare 45 min to 300 min). Xylopentaose and xylohexaose quickly maximized at 45 min in TM3-hydrolyzed xylan. However, the peak time of these two xylooligosaccharides was delayed to 120 min in TM1 and TM1 + TM2-hydrolyzed xylan samples.

From this analysis, it is known that *Cb*Xyn10C and *Cb*Cel48B also synergized intramolecularly in degrading xylan, manifested by higher amounts of shorter xylooligosaccharides released by TM3 during the early stage of hydrolysis (Fig. 3). The synergy is partially accounted for by the promiscuous activity of *Cb*Cel48B on xylan, which preferentially releases xylobiose and xylotriose from xylan (Additional file 2). As a typical endoxylanase, *Cb*Xyn10C randomly cleaved the xylan backbone and generated long xylooligosaccharides, which could then be converted into shorter ones by *Cb*Cel48B. Apparently, the proximity of *Cb*Xyn10C and *Cb*Cel48B due to co-existence within a single polypeptide further facilitated this coordinated hydrolysis, since no synergy was observed for incubation of xylan with the TM1/TM2 mixture.

In contrast to the strong synergy exhibited in initial hydrolysis of xylan, no synergy was found for *Cb*Xyn10C and *Cb*Cel48B, either inter- or intramolecularly, at the end of reactions. This paradox may be explained by a hypothesis that, with the time passing, *Cb*Cel48B was gradually inhibited by the increasing concentrations of xylose, xylobiose, and other xylooligosaccharides. Indeed, using xylotetraose as a model xylooligosaccharide substrate and xylobiose as a model inhibitor, it was demonstrated that degradation of xylotetraose by *Cb*Cel48B was inhibited by increasing amounts of xylobiose (from 320 to 6000 μM, Fig. 3d). In addition, the synergy observed only at the early stage of xylan hydrolysis implied that a comparably higher degree of polymerization for the substrates was critical for concerted action of the two enzymes. During hydrolysis, the length of the substrates was continuing to decrease, which attenuated the synergistic hydrolysis. Finally, with superior activity on xylose-configured oligo- and polysaccharide substrates, *Cb*Xyn10C tended to dominate the late stage of xylan hydrolysis. Therefore, at the end of reaction TM1, TM1 + TM2, and TM3 all demonstrated a nearly identical mode of action for xylan hydrolysis, all reflective of the mode of action of TM1 on xylan.

*Caldicellulosiruptor bescii* is a bacterium that grows by attaching to the surface of lignocellulose [9]. This special lifestyle ensures that sugars released from plant cell wall polysaccharides can be rapidly captured and assimilated by the bacterium. The precise local concentrations of xylose and xylooligosaccharides near *Cb*Xyn10C/Cel48B in its real living environment have not been determined yet. If the concentration of released oligosaccharides is not so high, *Cb*Cel48B (and its homologs *Cb*Cel48A and *Cb*Cel48C) will not be inhibited and can continue to synergize with *Cb*Xyn10C in hydrolyzing xylan. The faster hydrolysis of xylan by *Cb*Xyn10C and *Cb*Cel48B within the same polypeptide than acting individually in the early stage of hydrolysis could be beneficial for *C. bescii* to inhabit and outcompete other contester microbes on lignocellulose.

### The GH10 and GH48 catalytic domains displayed synergy in degrading cellulose, but not xylan, for complex lignocellulose corn straw

**Fig. 4:**
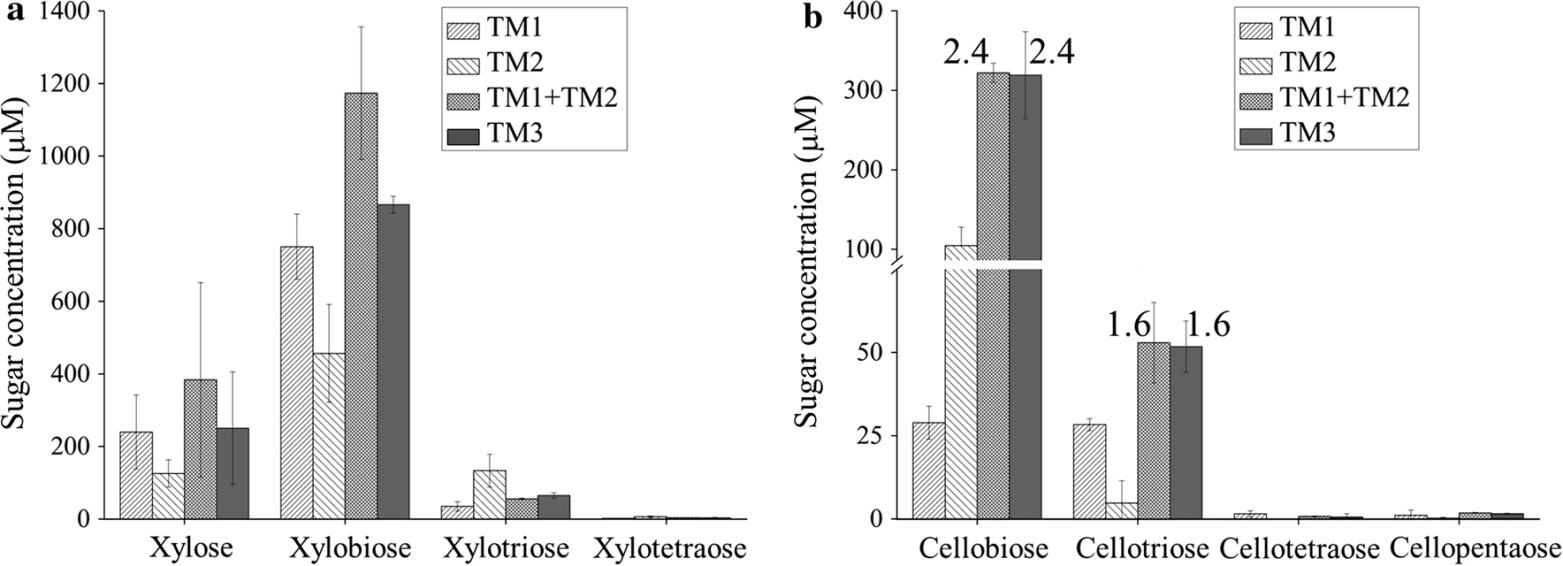
The GH10 and GH48 domains acted synergistically on cellulose hydrolysis when incubated with corn straw. Corn straw (5 mg/ml) was incubated with 1.5 μM each of truncation mutants, and the reaction was carried out at 75 °C in the McIlvaine buffer (pH 6.5) for 5 h. Released xylose and xylooligosaccharides (**a**) or cellooligosaccharides (**b**) as analyzed by HPAEC–PAD. The degrees of synergy in releasing cellobiose and cellotriose were labeled in **b**

*Caldicellulosiruptor bescii* is able to degrade and utilize complex and untreated lignocellulose switchgrass [30], during which *Cb*Xyn10C/Cel48B is highly expressed [10], pinpointing to the importance of this multimodular enzyme in assisting the bacterium to acquire energy from lignocellulose. Therefore, we determined the activity of TM3, a miniature version of *Cb*Xyn10C/Cel48B devoid of CBM3b-1, on steam explosion-treated corn straw, which has cellulose and xylan as the main components and compared its hydrolysis by TM1, TM2, and TM1 + TM2. The assay conditions were also set as 75 °C in the pH 6.5 McIlvaine buffer. It appeared that much less amounts of xylooligosaccharides were released from corn straw (Fig. 4a) when compared with those released from the pure model xylan substrate (Fig. 3). Xylotetraose and larger xylooligosaccharides were even not detected. This is likely due to the much higher recalcitrance of corn straw, which is a complex lignocellulose. There was no synergy for the GH10 and GH48 in degrading the xylan component (Fig. 4a) and this could be explained by the completed reaction with the xylan component, as observed for hydrolysis of pure xylan at 18 h (1080 min, Fig. 3). TM3 displayed even slightly lower activity compared to the TM1 + TM2 binary enzyme mixture. However, degradation of corn straw cellulose by these enzymes was similar to that for pure cellulose. TM1 + TM2 displayed degrees of synergy of 2.4 (for cellobiose) and 1.6 (for cellotriose) compared to using TM1 and TM2 alone (Fig. 4b). An identical extent of cellulose hydrolysis was observed for TM3 as compared to TM1 + TM2.

Taken together, our results demonstrated for the first time that the GH10 and GH48 catalytic domains, which are often simply recognized as xylanase and cellulase, respectively, can cooperate in hydrolyzing both cellulose and xylan. While synergy in xylan hydrolysis is limited to intramolecular domain–domain cooperation, that in cellulose degradation is not. The current findings will be helpful for understanding the physiology of *C. bescii* in capturing energy from the recalcitrant lignocellulose. The dual function (cellulase and xylanase) for both *Cb*Xyn10C and *Cb*Cel48B and the covalent linkage of these catalytic domains in a same polypeptide are both necessary determinants for maximal cooperation on multiple polysaccharide substrates. There are two more GH48 enzymes encoded by *C. bescii*, which are also the C-terminal components of multimodular glycoside hydrolases (CelA: GH9-CBM3c-CBM3b-CBM3b-GH48; CelF: GH74-CBM3b-CBM3b-GH48). Since *Cb*Cel48B are completely identical to the two GH48 domains in CelA and CelF, *Cb*Xyn10C can cooperate with the two GH48 domains in CelA and CelF, in part explaining why these multimodular proteins can synergize in hydrolyzing cellulose [16]. On the other hand, in addition to *Cb*Xyn10C/Cel48B, four out of the rest five multimodular enzymes have biochemically defined endoglucanase domains (GH9 in CelA and CelE, GH44 in CelB, and GH5 in CelD). The intermolecular synergy between *Cb*Cel48B and *Cb*Xyn10C in hydrolyzing cellulose suggests a possibility of synergistic effect of *Cb*Cel48B with these cellulase domains, which deserves further investigation.

The importance of GH48 cellulase to bacterial utilization of crystalline cellulose has long been addressed from the perspectives of genetics [31], metatranscriptomic [32], and genomic analyses [33]. In *Caldicellulosiruptor*, the special domain organization of GH10 and GH48 domains in a protein is not unique only for *Cb*Xyn10C/Cel48B. In the sequenced *Caldicellulosiruptor* genomes, the co-existence of *Cb*Xyn10C and *Cb*Cel48B homologs within a same polypeptide is also discovered for *C. kronotskyensis*, *C. naganoensis*, *C. danielii*, *C. changbaiensis*, and *C. morganii* in addition to *C. bescii* (Additional file 3) [33–35]. Notably, all these bacteria are efficient lignocellulose degraders. Therefore, these proteins might contribute significantly to enzymatic hydrolysis of cellulose and xylan by these bacteria.

Multimodular enzymes with two catalytic domains targeting different components of plant cell wall polysaccharides are rare in nature. However, there are still examples of such naturally occurring enzymes which simultaneously attack cellulose and xylan [36, 37], the xylan backbone and its ferulic acid side chain [38], or the pectin polygalacturonic acid backbone and its methyl-ester side chain [39]. It should also be noted that in sequenced microbial genomes or metagenomes there are enzymes predicted to bear multiple catalytic domains whose enzymatic properties are only computationally predicted but poorly biochemically characterized [40]. Due to the lack of knowledge about the substrate promiscuity of the catalytic domains in such enzymes, whether there is a synergy between the respective catalytic domains remains to be elucidated. Moreover, our findings pinpoint the importance of both the substrate promiscuity and the means of domain organization, which will shed light on designing new and robust multimodular glycoside hydrolases for efficient hydrolysis of plant cell wall polysaccharides.

## Conclusions

In this study, we discovered that the endoxylanase *Cb*Xyn10C and cellobiohydrolase *Cb*Cel48B from *C. bescii* can synergize in hydrolyzing both cellulose and xylan. The synergy was contributed by the primary activity of one catalytic domain in cooperation with the accessory activity of the other catalytic domain. Synergistic hydrolysis of xylan is dependent on covalent linkage of the two domains in the same polypeptide, while that of cellulose can take place both inter- and intramolecularly. The findings will be helpful for understanding the bacterial physiology of *C. bescii* regarding its energy capture and can also be used to guide design of new enzyme cocktails for efficient plant cell wall polysaccharides depolymerization.

## Methods

### Strains and plasmids

The pEASY-T3 plasmid and the *Escherichia coli* Trans1 strain (Transgen, Beijing, China) were used for gene cloning and plasmid propagation throughout the study. The pET-28a(+) plasmid (Merck, Darmstadt, Germany) and *E. coli* BL21(DE3) (Transgen, Beijing, China) were used for recombinant enzyme expression. The genomic DNA of *C. bescii* DSMZ 6725 was purchased from the Deutsche Sammlung von Mikro-organismen und Zellkulturen GmbH (https://www.dsmz.de/).

### Construction of the expression vectors

The DNA fragments encoding GH10-CBM3b (TM1) and CBM3b-GH48 (TM2) were amplified from the genomic DNA of *C. bescii* using gene-specific primers (for sequences see Additional file 4) and ligated to the EcoRI/XhoI (for TM1) or SacI/XhoI (for TM2)-restriction digested pET-28a(+) to obtain pET28-TM1 and pET28-TM2. The expression vector for GH10-CBM-GH48 (pET28-TM3) was constructed by ligating the DNA fragment encoding GH10 in-frame with the 5′ end of *CBM3b*-*GH48* in pET28-TM2. The integrity of the inserted DNA was verified by DNA sequencing.

### Expression and purification of the TM1, TM2 and TM3 recombinant proteins

The expression plasmids, pET28-TM1, pET28-TM2, and pET28-TM3, were individually transformed into the *E. coli* BL21 (DE3), which served as a host cell for expression of recombinant proteins. Expression and purification of TM1 was carried out as described by us previously [17]. For TM2 and TM3, the expression and purification were as the following. The recombinant strains were grown at 37 °C in the lysogeny broth (LB) containing 50 μg/ml of kanamycin. When the cell density reached an OD_600_ of 0.6, 0.8 mM final concentration of isopropyl-1-thio-β-d-galactopyranoside (IPTG) was added and the culture was continued at 37 °C for 4 h for inducing the recombinant protein. The bacterial cells were centrifuged and then re-suspended in a lysis buffer (20 mM Tris–HCl [pH 7.5], 500 mM NaCl) containing 1 mM of phenylmethylsulfonyl fluoride (PMSF). The cells were sonicated for cell wall disruption and centrifuged at 12,000*g* for 15 min. The supernatant was collected and applied to a 5 ml HisTrap HP column (GE Healthcare, Piscataway, NJ) for immobilized metal affinity chromatography (IMAC). The recombinant enzymes were eluted from the column by using the buffer (20 mM Tris–HCl [pH 7.5], 500 mM NaCl) containing gradient imidazole from 0 to 500 mM. The fractions containing targeted proteins were combined and concentrated by using a 50-ml ultrafiltration tube (Millipore, Bedford, MA). Next, the crude enzymes were individually loaded onto a 5-ml HiTrap Q column (GE Healthcare, Piscataway, NJ) for ion exchange purification using Buffer A (20 mM Tris–HCl [pH 8.0]) as the binding buffer and Buffer B (20 mM Tris–HCl [pH 8.0], 1 M NaCl) as the elution buffer, respectively. TM2 was finally passed through a HiPrep 16/60 Sephacryl S-100 column (GE Healthcare, Piscataway, NJ) and TM3 was passed through a Superose 12 10/300 GL (GE Healthcare, Piscataway, NJ) for further gel filtration purification. The fractions containing pure proteins were pooled.

### Assay of the specific enzyme activities

The specific activities of TM1, TM2, and TM3 on cellulose and xylan were determined by measuring the release of reducing sugars using the 2,5-dinitrosalicylic acid (DNS) method [41]. Briefly, appropriately diluted enzymes (10 nM each for measurement of the xylanase activity, and 1.5 μM for measurement of the cellulase activity) were individually incubated with beech wood xylan, sodium carboxymethyl cellulose (CMC), or Whatman No. 1 filter paper in a McIlvaine buffer (200 mM sodium phosphate, 100 mM sodium citrate, pH 6.5) at 85 °C for xylanase or 75 °C for cellulase activity. The reaction time was 10 min and 120 min for measurement of xylanase and cellulase activity, respectively. At the end of reaction, DNS was added and the mixture was incubated in boiling water for 5 min [41].

### Filter paper hydrolysis

For filter paper hydrolysis, 1.5 μM of TM1, TM2, TM1 + TM2 or TM3 was individually incubated with 5 mg/ml of Whatman No. 1 filter paper in the McIlvaine buffer (pH 6.5) at 75 °C for 5 h. The released reducing sugars were determined by using the DNS method or HPAEC–PAC (high-performance anion-exchange chromatography with pulsed amperometric detection, see below for details).

### Time-course analysis of xylan and xylotetraose hydrolysis

For time-course analysis of xylan hydrolysis, 10 nM of TM1, TM1 + TM2, or TM3 was incubated with 5 mg/ml of beech wood xylan in the McIlvaine buffer (pH 6.5) at 75 °C for 18 h. Samples were taken out at different time interval for HPAEC–PAD analysis. For xylotetraose hydrolysis, 1 mM of xylotetraose was incubated with 0.5 μM of *Cb*Cel48B in absence or presence of 320, 2000, and 6000 μM of xylobiose. The samples were also taken out periodically for HPAEC–PAD analysis.

### Corn straw hydrolysis

For corn straw hydrolysis, 1.5 μM of TM1, TM2, TM1 + TM2, or TM3 was individually incubated with 5 mg/ml of stream explosion pretreated corn straw in the McIlvaine buffer (pH 6.5) at 75 °C for 5 h. The released reducing sugars were determined by using HPAEC–PAC.

### HPAEC–PAD

After incubation, the reaction mixture was boiled for 10 min to terminate the reaction, centrifuged at 12,000*g* for 10 min, and then filtered through a Nanosep centrifugal 3 K device (Pall, New York, NY) to remove the enzymes. Appropriately diluted reaction products were analyzed by HPAEC–PAD, which was equipped with a CarboPac PA100 guard column (4 × 50 mm), an analytical column (4 × 250 mm), and a pulsed amperometric detector ICS-5000 (Dionex, Sunnyvale, CA). The flow rate of the mobile phase was 1 ml/min at ambient temperature (22 °C). Glucose and cellooligosaccharides (cellobiose to cellohexaose), xylose and xylooligosaccharides (xylobiose to xylohexaose) (Megazyme, Wicklow, Ireland) were used as standards.

## Supplementary information

**Additional file 1.** Thermostability of the truncation mutants.

**Additional file 2.** Xylan hydrolysis by different concentrations of TM2, as analyzed by HPAEC–PAD.

**Additional file 3.** Amino acid sequence alignment of *Cb*Xyn10C/Cel48B with its homologs in the *Caldicellulosiruptor* genus. The GenBank accession numbers for the homologs in *C. kronotskyensis*, *C. naganoensis*, *C. danielii*, *C. changbaiensis*, and *C. morganii* are WP_013429870.1, WP_083943509.1, WP_045175321.1, WP_127352229.1, and WP_082054594.1, respectively.

**Additional file 4.** Primers used in this study.

## Data Availability

All data supporting the conclusions of this article are included within the manuscript and additional files.

## Abbreviations

GH: glycoside hydrolase
CBM: carbohydrate-binding module
PCWP: plant cell wall polysaccharides
LPMO: lytic polysaccharide monooxygenases
CMC: sodium carboxymethyl cellulose
HPAEC–PAD: high-performance anion-exchange chromatography with pulsed amperometric detection
IPTG: isopropyl-1-thio-β-d-galactopyranoside
PMSF: phenylmethylsulfonyl fluoride
IMAC: immobilized metal affinity chromatography

## References

[1] Doi RH, Kosugi A (2004). Cellulosomes: plant-cell-wall-degrading enzyme complexes. Nat Rev Microbiol.

[2] Menon V, Rao M (2012). Trends in bioconversion of lignocellulose: biofuels, platform chemicals & biorefinery concept. Prog Energy Combust Sci.

[3] Somerville C, Youngs H, Taylor C, Davis SC, Long SP (2010). Feedstocks for lignocellulosic biofuels. Science.

[4] Klein-Marcuschamer D, Oleskowicz-Popiel P, Simmons BA, Blanch HW (2012). The challenge of enzyme cost in the production of lignocellulosic biofuels. Biotechnol Bioeng.

[5] Xue XL, Wu YL, Qin X, Ma R, Luo HY, Su XY, Yao B (2016). Revisiting overexpression of a heterologous beta-glucosidase in *Trichoderma reesei*: fusion expression of the *Neosartorya fischeri* Bgl3A to cbh1 enhances the overall as well as individual cellulase activities. Microb Cell Fact.

[6] Zhang J, Zhong Y, Zhao X, Wang T (2010). Development of the cellulolytic fungus *Trichoderma reesei* strain with enhanced beta-glucosidase and filter paper activity using strong artificial cellobiohydrolase 1 promoter. Bioresour Technol.

[7] Hemsworth GR, Johnston EM, Davies GJ, Walton PH (2015). Lytic polysaccharide monooxygenases in biomass conversion. Trends Biotechnol.

[8] Bischof RH, Ramoni J, Seiboth B (2016). Cellulases and beyond: the first 70 years of the enzyme producer *Trichoderma reesei*. Microb Cell Fact.

[9] Dam P, Kataeva I, Yang SJ, Zhou FF, Yin YB, Chou WC, Poole FL, Westpheling J, Hettich R, Giannone R (2011). Insights into plant biomass conversion from the genome of the anaerobic thermophilic bacterium *Caldicellulosiruptor bescii* DSM 6725. Nucleic Acids Res.

[10] Lochner A, Giannone RJ, Rodriguez M, Shah MB, Mielenz JR, Keller M, Antranikian G, Graham DE, Hettich RL (2011). Use of label-free quantitative proteomics to distinguish the secreted cellulolytic systems of *Caldicellulosiruptor bescii* and *Caldicellulosiruptor obsidiansis*. Appl Environ Microbiol.

[11] Ye L, Su X, Schmitz GE, Moon YH, Zhang J, Mackie RI, Cann IK (2012). Molecular and biochemical analyses of the GH44 module of CbMan5B/Cel44A, a bifunctional enzyme from the hyperthermophilic bacterium *Caldicellulosiruptor bescii*. Appl Environ Microbiol.

[12] Zverlov V, Mahr S, Riedel K, Bronnenmeier K (1998). Properties and gene structure of a bifunctional cellulolytic enzyme (CelA) from the extreme thermophile ‘*Anaerocellum thermophilum*’ with separate glycosyl hydrolase family 9 and 48 catalytic domains. Microbiology.

[13] Yi Z, Su X, Revindran V, Mackie RI, Cann I (2013). Molecular and biochemical analyses of CbCel9A/Cel48A, a highly secreted multi-modular cellulase by *Caldicellulosiruptor bescii* during growth on crystalline cellulose. PLoS ONE.

[14] Brunecky R, Alahuhta M, Xu Q, Donohoe BS, Crowley MF, Kataeva IA, Yang SJ, Resch MG, Adams MW, Lunin VV (2013). Revealing nature’s cellulase diversity: the digestion mechanism of *Caldicellulosiruptor bescii* CelA. Science.

[15] Young J, Chung D, Bomble YJ, Himmel ME, Westpheling J (2014). Deletion of *Caldicellulosiruptor bescii* CelA reveals its crucial role in the deconstruction of lignocellulosic biomass. Biotechnol Biofuels.

[16] Conway JM, McKinley BS, Seals NL, Hernandez D, Khatibi PA, Poudel S, Giannone RJ, Hettich RL, Williams-Rhaesa AM, Lipscomb GL (2017). Functional analysis of the Glucan Degradation Locus (GDL) in *Caldicellulosiruptor bescii* reveals essential roles of component glycoside hydrolases in plant biomass deconstruction. Appl Environ Microbiol.

[17] Xue XL, Wang R, Tu T, Shi PJ, Ma R, Luo HY, Yao B, Su XY (2015). The N-Terminal GH10 domain of a multimodular protein from *Caldicellulosiruptor bescii* is a versatile xylanase/beta-glucanase that can degrade crystalline cellulose. Appl Environ Microbiol.

[18] Conway JM, Crosby JR, Hren AP, Southerland RT, Lee LL, Lunin VV, Alahuhta P, Himmel ME, Bomble YJ, Adams MWW, Kelly RM (2018). Novel multidomain, multifunctional glycoside hydrolases from highly lignocellulolytic *Caldicellulosiruptor* species. AIChE J.

[19] Conway JM, Crosby JR, McKinley BS, Seals NL, Adams MWW, Kelly RM (2018). Parsing in vivo and in vitro contributions to microcrystalline cellulose hydrolysis by multidomain glycoside hydrolases in the *Caldicellulosiruptor bescii* secretome. Biotechnol Bioeng.

[20] Chu Y, Tu T, Penttinen L, Xue X, Wang X, Yi Z, Gong L, Rouvinen J, Luo H, Hakulinen N (2017). Insights into the roles of non-catalytic residues in the active site of a GH10 xylanase with activity on cellulose. J Biol Chem.

[21] Brunecky R, Chung D, Sarai NS, Hengge N, Russell JF, Young J, Mittal A, Pason P, Vander Wall T, Michener W (2018). High activity CAZyme cassette for improving biomass degradation in thermophiles. Biotechnol Biofuels.

[22] Riedel K, Bronnenmeier K (1998). Intramolecular synergism in an engineered exo-endo-1,4-beta-glucanase fusion protein. Mol Microbiol.

[23] Poudel S, Giannone RJ, Basen M, Nookaew I, Poole FL, Kelly RM, Adams MWW, Hettich RL (2018). The diversity and specificity of the extracellular proteome in the cellulolytic bacterium *Caldicellulosiruptor bescii* is driven by the nature of the cellulosic growth substrate. Biotechnol Biofuels.

[24] Saqib AAN, Whitney PJ (2011). Differential behaviour of the dinitrosalicylic acid (DNS) reagent towards mono- and di-saccharide sugars. Biomass Bioenergy.

[25] Lee LL, Blumer-Schuette SE, Izquierdo JA, Zurawski JV, Loder AJ, Conway JM, Elkins JG, Podar M, Clum A, Jones PC (2018). Genus-wide assessment of lignocellulose utilization in the extremely thermophilic genus *Caldicellulosiruptor* by genomic, pangenomic, and metagenomic analyses. Appl Environ Microbiol.

[26] Wang R, Gong L, Xue XL, Qin X, Ma R, Luo HY, Zhang YJ, Yao B, Su XY (2016). Identification of the C-Terminal GH5 domain from *Cb*Cel9B/Man5A as the first glycoside hydrolase with thermal activation property from a multimodular bifunctional enzyme. PLoS ONE.

[27] Bok JD, Yernool DA, Eveleigh DE (1998). Purification, characterization, and molecular analysis of thermostable cellulases CelA and CelB from *Thermotoga neapolitana*. Appl Environ Microbiol.

[28] Park JI, Kent MS, Datta S, Holmes BM, Huang ZH, Simmons BA, Sale KL, Sapra R (2011). Enzymatic hydrolysis of cellulose by the cellobiohydrolase domain of CelB from the hyperthermophilic bacterium *Caldicellulosiruptor saccharolyticus*. Bioresour Technol.

[29] Su XY, Mackie RI, Cann IKO (2012). Biochemical and mutational analyses of a multidomain cellulase/mannanase from *Caldicellulosiruptor bescii*. Appl Environ Microbiol.

[30] Yang SJ, Kataeva I, Wiegel J, Yin YB, Dam P, Xu Y, Westpheling J, Adams MWW (2010). Classification of ‘*Anaerocellum thermophilum*’ strain DSM 6725 as *Caldicellulosiruptor bescii* sp. nov. Int J Syst Evol Microbiol.

[31] Olson DG, Tripathi SA, Giannone RJ, Lo J, Caiazza NC, Hogsett DA, Hettich RL, Guss AM, Dubrovsky G, Lynd LR (2010). Deletion of the Cel48S cellulase from *Clostridium thermocellum*. Proc Natl Acad Sci USA.

[32] Dai X, Tian Y, Li J, Luo Y, Liu D, Zheng H, Wang J, Dong Z, Hu S, Huang L (2015). Metatranscriptomic analyses of plant cell wall polysaccharide degradation by microorganisms in the cow rumen. Appl Environ Microbiol.

[33] Lee LL, Blumer-Schuette SE, Izquierdo JA, Zurawski JV, Loder AJ, Conway JM, Elkins JG, Podar M, Clum A, Jones PC (2018). Genus-wide assessment of lignocellulose utilization in the extremely thermophilic genus *Caldicellulosiruptor* by genomic, pangenomic, and metagenomic analyses. Appl Environ Microbiol..

[34] Bing W, Wang H, Zheng B, Zhang F, Zhu G, Feng Y, Zhang Z (2015). *Caldicellulosiruptor changbaiensis* sp. nov., a cellulolytic and hydrogen-producing bacterium from a hot spring. Int J Syst Evol Microbiol.

[35] Miroshnichenko ML, Kublanov IV, Kostrikina NA, Tourova TP, Kolganova TV, Birkeland NK, Bonch-Osmolovskaya EA (2008). *Caldicellulosiruptor kronotskyensis* sp. nov. and *Caldicellulosiruptor hydrothermalis* sp. nov., two extremely thermophilic, cellulolytic, anaerobic bacteria from Kamchatka thermal springs. Int J Syst Evol Microbiol.

[36] Rashamuse KJ, Visser DF, Hennessy F, Kemp J, Roux-van der Merwe MP, Badenhorst J, Ronneburg T, Francis-Pope R, Brady D (2013). Characterisation of two bifunctional cellulase-xylanase enzymes isolated from a bovine rumen metagenome Library. Curr Microbiol.

[37] Yuan SF, Wu TH, Lee HL, Hsieh HY, Lin WL, Yang B, Chang CK, Li Q, Gao J, Huang CH (2015). Biochemical characterization and structural analysis of a bifunctional cellulase/xylanase from *Clostridium thermocellum*. J Biol Chem.

[38] Dodd D, Kocherginskaya SA, Spies MA, Beery KE, Abbas CA, Mackie RI, Cann IK (2009). Biochemical analysis of a beta-d-xylosidase and a bifunctional xylanase-ferulic acid esterase from a xylanolytic gene cluster in *Prevotella ruminicola* 23. J Bacteriol.

[39] Tu T, Bai Y, Luo H, Ma R, Wang Y, Shi P, Yang P, Meng K, Yao B (2014). A novel bifunctional pectinase from *Penicillium oxalicum* SX6 with separate pectin methylesterase and polygalacturonase catalytic domains. Appl Microbiol Biotechnol.

[40] Talamantes D, Biabini N, Dang H, Abdoun K, Berlemont R (2016). Natural diversity of cellulases, xylanases, and chitinases in bacteria. Biotechnol Biofuels.

[41] Miller GL (1959). Use of dinitrosalicylic acid reagent for determination of reducing sugar. Anal Chem.

