# Supplementary material for: The GH10 and GH48 dual-functional catalytic domains from a multimodular glycoside hydrolase synergize in hydrolyzing both cellulose and xylan
Source: Biotechnol Biofuels. 2019 Dec 3;12:279. doi: 10.1186/s13068-019-1617-2 (PMC6892212; doi:10.1186/s13068-019-1617-2)

**Additional files**

**Additional file 1.** Thermostability of the truncation mutants. The TM1, TM2, and TM3 mutant enzymes were individually incubated at 75ºC for 18 h. At different time points (0.5 h, 1 h, 2 h, 4 h, 8 h, and 18 h), samples were taken out and assayed for residual activity. The activity of TM1 was determined using 1% xylan as the substrate in a 10-min assay in 100 mM McIlvaine buffer (pH6.5). The reducing sugar was measured using the DNS method and the absorbance was monitored at 540 nm. For TM2 and TM3, 4-methylumbelliferyl β-D-cellobiose (MUC) was used as the substrate. The assay was carried out at 75ºC for 15 min. Then equal volume of Na_2_CO_3_ (1 M) was added to terminate the reaction. The absorbance was measured at 370 nm. The relative activities at different time points were calculated by dividing the values against that at time 0, which was set as 100%.


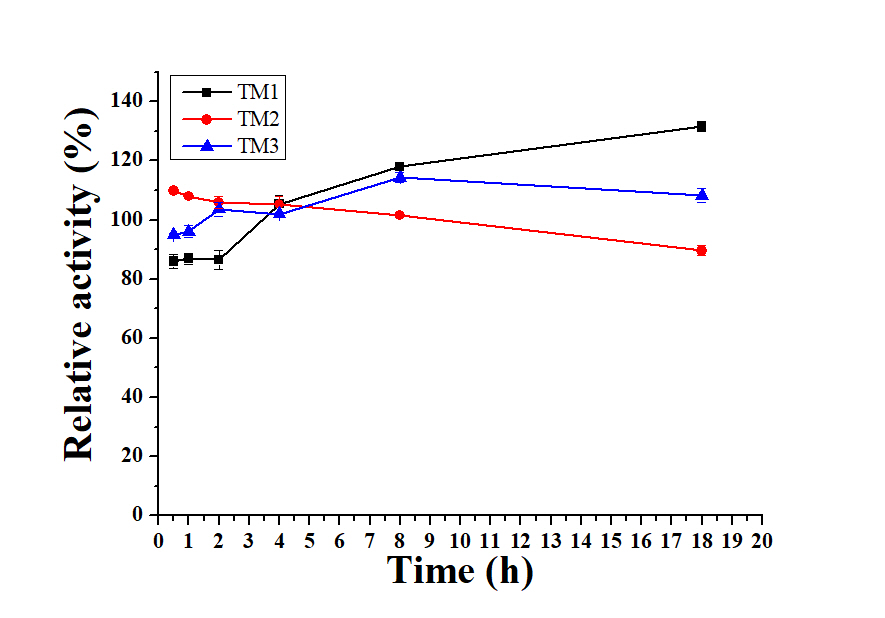

Supplement: Supplementary file 1 — Additional file 1. Thermostability of the truncation mutants. [file 13068_2019_1617_MOESM1_ESM.docx]
