# Supplementary material for: The GH10 and GH48 dual-functional catalytic domains from a multimodular glycoside hydrolase synergize in hydrolyzing both cellulose and xylan
Source: Biotechnol Biofuels. 2019 Dec 3;12:279. doi: 10.1186/s13068-019-1617-2 (PMC6892212; doi:10.1186/s13068-019-1617-2)
Supplement: Supplementary file 2 — Additional file 2. Xylan hydrolysis by different concentrations of TM2, as analyzed by HPAEC–PAD. [file 13068_2019_1617_MOESM2_ESM.docx]

**Additional file 2:** Xylan hydrolysis by different concentrations of TM2, as analyzed by HPAEC-PAD.
