# Supplementary material for: The GH10 and GH48 dual-functional catalytic domains from a multimodular glycoside hydrolase synergize in hydrolyzing both cellulose and xylan
Source: Biotechnol Biofuels. 2019 Dec 3;12:279. doi: 10.1186/s13068-019-1617-2 (PMC6892212; doi:10.1186/s13068-019-1617-2)

**Additional file 3:** Amino acid sequence alignment of *Cb*Xyn10C/Cel48B with its homologs in the *Caldicellulosiruptor* genus. The GenBank accession numbers for the homologs in *C. kronotskyensis*, *C. naganoensis*, *C. danielii*, *C. changbaiensis*, and *C. morganii* are WP_013429870.1, WP_083943509.1, WP_045175321.1, WP_127352229.1, and WP_082054594.1, respectively. The sequences start from the GH10 xylanase with signal peptide removed and the numbers are for *Cb*Xyn10C/Cel48B labeling.


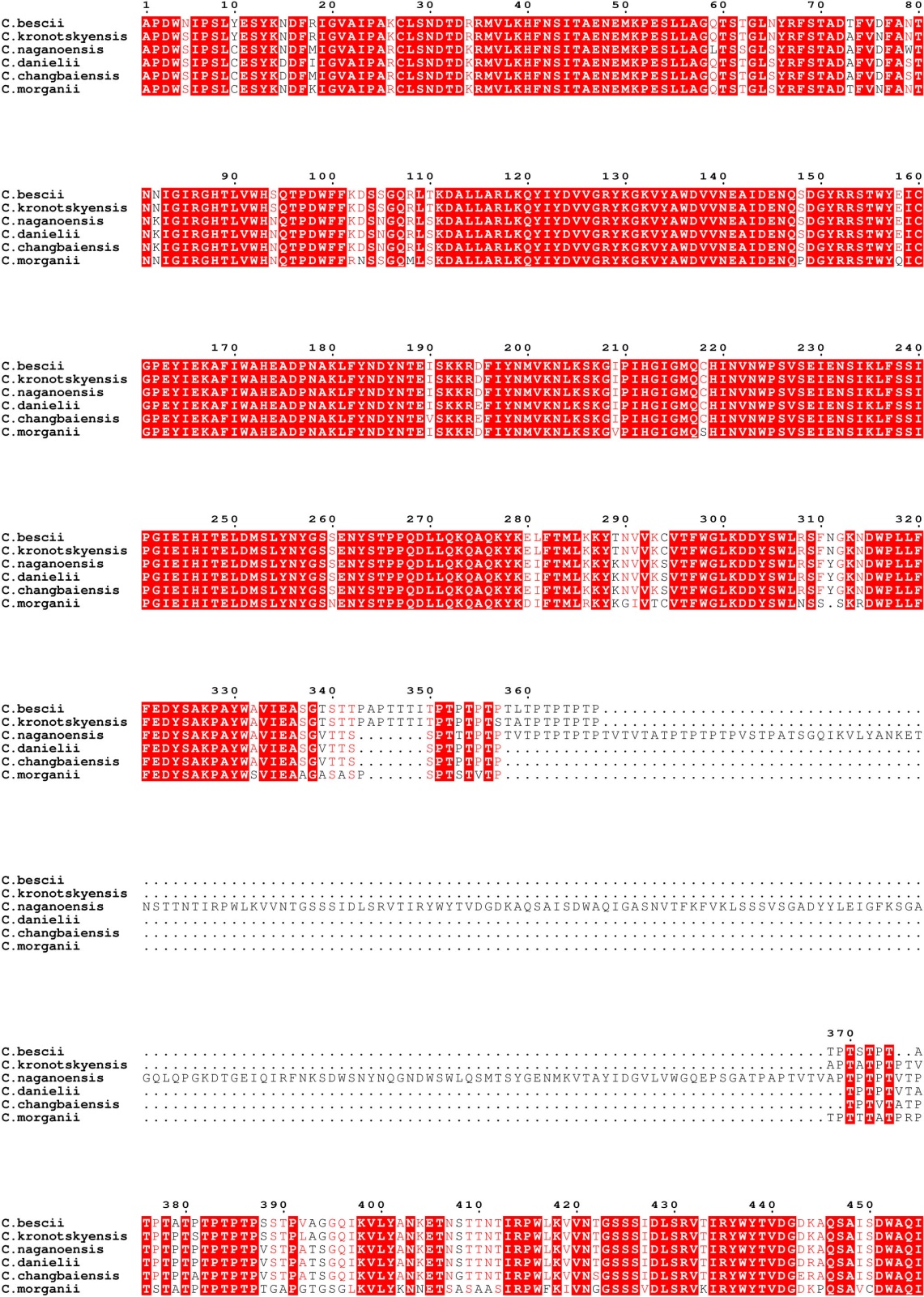


**Additional file 3:** Amino acid sequence alignment of *Cb*Xyn10C/Cel48B with its homologs in the *Caldicellulosiruptor* genus (cont’d).


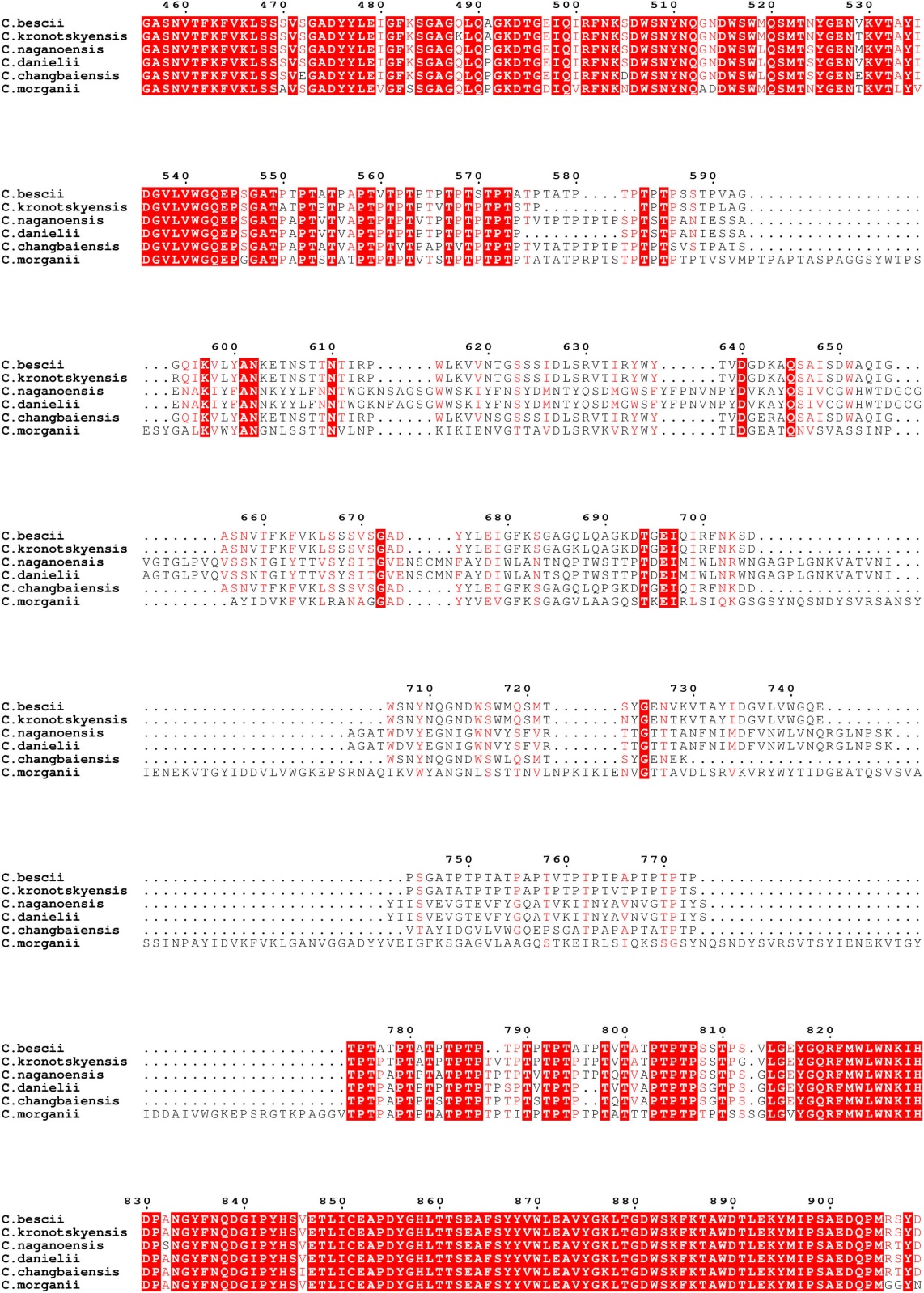


**Additional file 3:** Amino acid sequence alignment of *Cb*Xyn10C/Cel48B with its homologs in the *Caldicellulosiruptor* genus (cont’d).


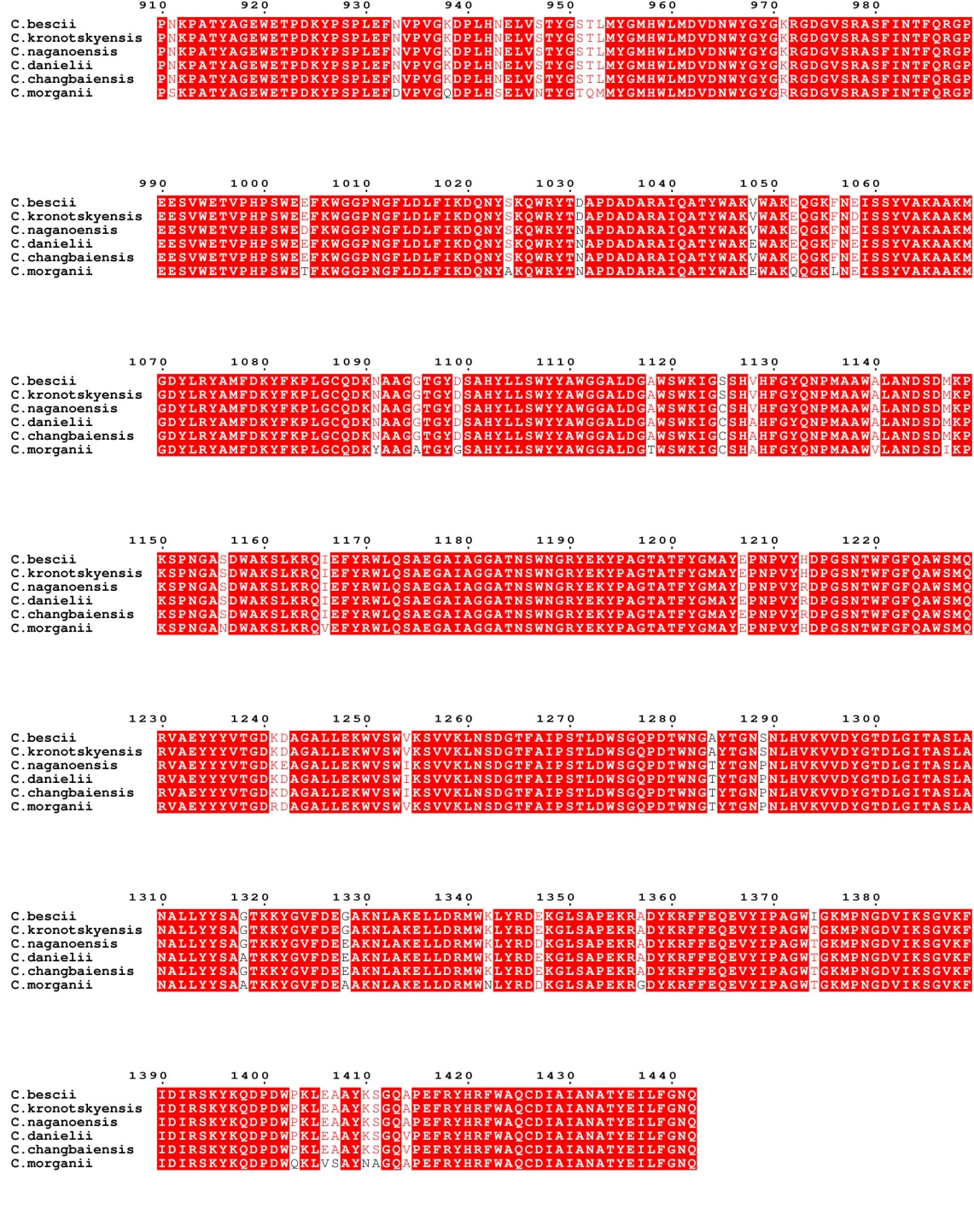

Supplement: Supplementary file 3 — Additional file 3. Amino acid sequence alignment of CbXyn10C/Cel48B with its homologs in the Caldicellulosiruptor genus. The GenBank accession numbers for the homologs in C. kronotskyensis, C. naganoensis, C. danielii, C. changbaiensis, and C. morganii are WP_013429870.1, WP_083943509.1, WP_045175321.1, WP_127352229.1, and WP_082054594.1, respectively. [file 13068_2019_1617_MOESM3_ESM.docx]
