# Supplementary material for: The GH10 and GH48 dual-functional catalytic domains from a multimodular glycoside hydrolase synergize in hydrolyzing both cellulose and xylan
Source: Biotechnol Biofuels. 2019 Dec 3;12:279. doi: 10.1186/s13068-019-1617-2 (PMC6892212; doi:10.1186/s13068-019-1617-2)
Supplement: Supplementary file 4 — Additional file 4. Primers used in this study. [file 13068_2019_1617_MOESM4_ESM.docx]

**Additional file 4. Primers used in this study.**

| **Primer^1^** | **Sequence^2^** | **Usage** |
| --- | --- | --- |
| TM1_s | GGGAATTCCCTGACTGGAACATTCCAAGTTTATATG | For TM1 |
| TM1_a | GGCTCGAGGGTGTCGGTGTCACTGTCGGTG |  |
| TM2_s  TM2_a | GCGGGAGCTCGGACAGATAAAGGTATTGTATGCTA  GCGGCTCGAGGGTACCTTGATTGCCAAACAGTATTTCATAT | For TM2 |
| TM3_s  TM3_a | GGATCCCCTGACTGGAACATTCCAAGTTTATATG  GCGGGAGCTCTCCAGCTACAGGTGTTGAAGAAGG | For TM3 |

^1^Forward and reverse primes are abbreviated as s and a, respectively.

^2^ Restriction sites are underlined.
